# Flowers and Leaves of *Artemisia absinthium* and *Artemisia annua* Phytochemical Characterization, Anti-Inflammatory, Antioxidant, and Anti-Proliferative Activities Evaluation

**DOI:** 10.3390/plants14071029

**Published:** 2025-03-26

**Authors:** Mădălina Țicolea, Raluca Maria Pop, Marcel Pârvu, Lia-Oxana Usatiuc, Ana Uifălean, Dalina Diana Pop, Eva Fischer-Fodor, Floricuța Ranga, Crina Claudia Rusu, Adriana Florinela Cătoi, Francisco Palma-Garcia, Luciana-Mădălina Gherman, Alina Elena Pârvu

**Affiliations:** 1 Department of Morpho-Functional Sciences, Discipline of Pathophysiology, “Iuliu Haţieganu” University of Medicine and Pharmacy, 400012 Cluj-Napoca, Romania; madalina.ticolea@umfcluj.ro (M.Ț.); lia.usatiuc@umfcluj.ro (L.-O.U.); uifaleanana@gmail.com (A.U.); adriana.catoi@umfcluj.ro (A.F.C.); parvualinaelena@umfcluj.ro (A.E.P.); 2 Department of Morpho-Functional Sciences, Discipline of Pharmacology, Toxicology and Clinical Pharmacology, “Iuliu Haţieganu” University of Medicine and Pharmacy, 400337 Cluj-Napoca, Romania; 3 Department of Biology, Babes-Bolyai University, 400015 Cluj-Napoca, Romania; marcel.parvu@ubbcluj.ro; 4 Department of Morpho-Functional Sciences, Discipline of Anatomy, “Iuliu Haţieganu” University of Medicine and Pharmacy, 400012 Cluj-Napoca, Romania; diana.pop@umfcluj.ro; 5 Tumor Biology Department, The Oncology Institute I. Chiricuță, 400015 Cluj-Napoca, Romania; fischer.eva@iocn.ro; 6 Food Science and Technology, Department of Food Science, University of Agricultural Science and Veterinary Medicine Cluj-Napoca, 400372 Cluj-Napoca, Romania; floricutza_ro@yahoo.com; 7 Department of Nephrology, “Iuliu Haţieganu” University of Medicine and Pharmacy, 400012 Cluj-Napoca, Romania; claudia.rusu@umfcluj.ro; 8 “Mihai Manasia” Nephrology and Dialysis Clinic, County Emergency Clinical Hospital Cluj, 400347 Cluj-Napoca, Romania; 9 Faculty of Medicine, University of Panama, Panama City 64460, Panama; francisco.palma@up.ac.pa; 10 Experimental Center, “Iuliu Haţieganu” University of Medicine and Pharmacy Cluj-Napoca, 400349 Cluj-Napoca, Romania; luciana.gherman@umfcluj.ro

**Keywords:** *A. absinthium*, *A. annua*, polyphenols, inflammation, oxidative stress, antiproliferative

## Abstract

This study investigates the phytochemical composition, anti-inflammatory, antioxidant, and antiproliferative activities of *A. absinthium* and *A. annua* flowers and leaf ethanol extracts in acute rat inflammation model. Polyphenolic compounds were analyzed quantitatively (total phenolic (TPC) and total flavonoids (TFCs)) and qualitatively by HPLC-ESI MS analysis. The antioxidant activity was evaluated in vitro (by DPPH, FRAP, H_2_O_2_, and NO scavenging tests), and in vivo (by total oxidative status (TOS), total antioxidant capacity (TAC), oxidative stress index (OSI), and key oxidative damage markers). Inflammation was evaluated via nuclear factor-kB-p65 (NfkB-p65), and canonical NLRP3 inflammasome activation (with IL-1β, IL-18, caspase-1, and gasdermin D). The antiproliferative activity against human ovarian tumor cells (A2780cis, OVCAR-3, and OAW-42) was evaluated by the MTT assay, focusing on the modulation of multidrug resistance (MDR) pumps and the PARP-1 enzyme. Liver and renal toxicity were tested by measuring transaminases (ALT and AST), creatinine, and urea. The study results indicated that *A. absinthium* and *A. annua* flowers and leaf ethanol extracts have rich polyphenol content and moderate in vitro antioxidant activity. Tested extracts display an important antiproliferative activity against the ovarian tumor cell lines A2780cis, OVCAR-3, and OAW-42 based on chemoresistance countering and apoptotic mechanisms. There were differences related to the cell type and plant extract type. The tested plant extracts had significant and dose-dependent in vivo anti-inflammatory and antioxidant activity, with the *A. annua* flowers extract having the lowest efficiency. The anti-inflammatory and antioxidant activity biomarkers correlated with the extracts’ chemical composition. There was no inflammation-induced hepatotoxicity, but renal dysfunction was associated. Only AANL improved the renal function. These results can be used to design and develop remedies with combined anti-inflammatory, antioxidant, and anti-proliferative activities.

## 1. Introduction

For centuries, phytotherapy has been an important part of traditional medicine [1]. The *Artemisia genus* (Asteraceae), which bears the name of Artemis, the Greek goddess of the hunt, the wilderness, wild animals, nature, vegetation, childbirth, childcare, and chastity, is one of the most widely dispersed genera. The genus *Artemisia,* comprising more than 400 species of *Artemisia* plants has a worldwide distribution, especially in Asia, Europe, and North America [2]. Its wide distribution and rich composition of bioactive compounds contributed not only to its intensive use as a traditional medicine but also as edible plants, spices, or beverages. *Artemisia* remedies of traditional medicine have been applied in treating many diseases [3]. For example, *A. afra* Jacq. ex Willd. has anti-inflammatory effects, and it is used to treat fever, coughs, colds, malaria, influenza, and diabetes [4]. *A. dracunculus* L. has anti-diabetic and antioxidant properties and is used to treat wounds [5,6] while *A. vulgaris* L. is used as an anticancer, hepatoprotective, antiepileptic, antimalarial, and insecticidal agent. *A. scoparia*, besides its use as a spider bites treatment, was used as a diuretic, choleretic, and also to treat hepatitis, gastritis, ulcers, jaundice, itch, and asthma [7].

Moreover, the discovery of the antimalarial compound artemisinin by the Chinese scientist Youyou Tu, for which she received the 2015 Nobel Prize in Physiology or Medicine, reinvigorated the studies on *Artemisia* plants [3]. Scientific research on the plants from the *Artemisia* genus has found a complex composition and confirmed to existence of many pharmacological effects [8,9,10,11]. Numerous phytoconstituents, including essential oils, amino acids, terpenoids, alkaloids, tannins, quinines, flavonoids, and glycosides, were identified in different profiles and concentrations, which correlates with ecological and environmental factors, genetic origin, harvesting season, and harvesting technique, as well as extraction and processing techniques [12,13]. Among them, phenolic compounds exhibit diverse pharmacological properties, including antioxidant, anti-inflammatory, antimicrobial, and anticancer activities [7]. In *Artemisia* species, phenolic acids, like gallic acid, vanillic acid, caffeic acid, ferulic acid, sinapic acid, gentisic acid, and chlorogenic acid, and flavonoids, like catechin, epicatechin, rutin, quercetin, luteolin, isoquercitrin, quercetin, kaempferol, and apigenin, were reported as the main bioactive antioxidant phenolic compounds [2,14]. Accordingly, preclinical and clinical data indicate that the most extensively researched biological activity of these species includes the antioxidant, anti-inflammatory, anticancer, antidiabetic, antimalarial, neuroprotective, and hepatoprotective properties [3]. Among existing *Artemisia* species, *A. absinthium* and *A. annua* are the most famous. Their rich composition in phenolic compounds contributes to their pharmacological activities, particularly their antioxidant, anti-inflammatory, and anticancer effects [3].

In inflammation, inflammatory reactions are associated with immune system activation, which is triggered by infections and tissue injuries to protect organisms and restore tissue homeostasis. In the acute phase, vascular permeability is increased and neutrophil infiltration by paracellular and transcellular modes occurs [15,16]. In the interstitial tissue, recruited neutrophils are activated by cytokines and chemokines through phagocytosis, proteases release, reactive oxygen species (ROS) production, and the formation of neutrophils extracellular traps (NETs) to eliminate pathogens or tissue debris. Moreover, active neutrophils will release new cytokines and chemokines that will further modulate the inflammatory response and influence adaptive immunity. Excessive or deficient activation of all these mechanisms can turn an acute local inflammatory change into a case of systemic inflammation, chronic inflammation, autoimmunity, or cancer [15,17]. Further, chronic inflammation is associated with excessive ROS production and oxidative stress (OS), increased release of pro-inflammatory cytokines, and inflammasome activation. Oxidative stress occurs when ROS formation exceeds the buffering capacity of the endogenous antioxidant mechanisms, leading to DNA mutations, the oxidation of biomacromolecules, cells, and tissues, and finally injury leading to cellular damage [18].

Moreover, ROS and inflammatory cytokines can activate oncogenic signaling pathways, including MAPK, PI3K/AKT, and JAK/STAT, facilitating tumorigenesis [19]. Persistent OS and inflammation contribute to both epithelial-to-mesenchymal transition (EMT) and immune evasion, allowing cancer cells to spread and evade immune surveillance [20].

Given their multitargeted mechanisms of action, phenolic compounds from *A. absinthium* and *A. annua* represent promising candidates for drug discovery and the development of novel therapeutic strategies. Investigating their pharmacological potential could lead to the identification of new bioactive molecules that may be useful for the prevention and treatment of chronic diseases associated with oxidative stress, inflammation, and cancer progression.

Due to the large variability of bioactive compounds reported in the literature, the present study aimed to evaluate the phenolic compounds’ phytochemical composition of *A. absinthium* and *A. annua* flowers and leaf ethanol extracts, and their anti-inflammatory, antioxidant, and antiproliferative activities in turpentine-oil-induced rat inflammation. The study of the anti-inflammatory, antioxidant, and anticancer properties of *Artemisia* species is of significant scientific and therapeutic interest, as the contained phenolic compounds were reported to influence the pathophysiology behind multiple disorders, including cardiovascular diseases, neurodegenerative conditions, metabolic syndromes, and cancer specifically by modulating inflammation, oxidative stress, and tumor cell proliferation [3,21,22,23,24].

## 2. Results

### 2.1. Quantitative Phytochemical Analysis

*A. absinthium* and *A. annua* flower and leaf ethanol extracts TPC and TFC, are depicted in Table 1. In the *A. absinthium* flowers, the amount of extracted TPC was higher than in the *A. absinthium* leaf extract, but the difference was not significant (*p* > 0.05). In the case of *A. annua* flowers, the amount of extracted TPC was higher than in the *A. annua* leaf extract too, but the difference was more significant (*p* < 0.01). The ethanol extract from *A. absinthium* flowers had a lower TPC content than that of the *A. annua* flower ethanol extract (*p* < 0.01), and the ethanol extract from *A. absinthium* leaves had a higher TPC content than that of the *A. annua* leaf ethanol extract (*p* < 0.05) (Table 1).

TFC analysis found that, for both plants, the flower extracts had a significantly higher concentration of flavonoids than the leaf extracts (*p* < 0.01). The ethanol extract from *A. absinthium* flowers had a lower TFC content than that of the *A. annua* flowers (*p* < 0.01), and the ethanol extract from *A. absinthium* leaves had a lower TFC content than that of the *A. annua* leaves ethanol extract (*p* < 0.01) (Table 1).

### 2.2. Qualitative Phytochemical Analysis

**Figure 1:**
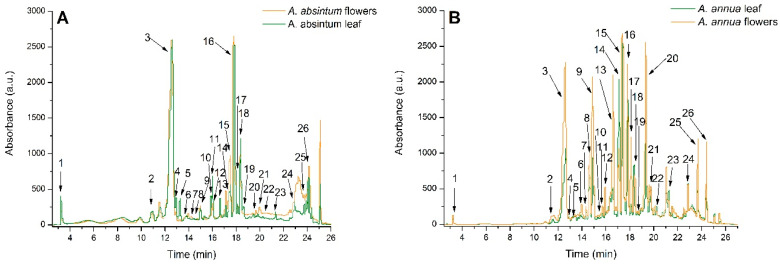
HPLC chromatogram of phenolic compounds identified in (**A**) *A. absinthium* flower ethanol extract and *A. absinthium* leaf ethanol extract, and in (**B**) *A. annua* flower ethanol extract and *A. absinthium* leaf ethanol extract. The peak identification is provided in Table 2.

The HPLC-ESI MS analysis identified 26 phenolic compounds in both the flowers and leaves of *A. absinthium* and *A. annua*. Compounds belonging to the hydroxycinnamic acids subclass represented 91.6% and 94.7% of the total phenolics in *A. absinthium* flowers and leaves, respectively, and 89.6% and 85.2% in *A. annua* flowers and leaves, respectively. Next, flavonols were present at levels of 5.05%, 4.32%, 5.21%, and 11.13% in both the flowers and leaves of *A. absinthium* and *A. annua*, respectively. Other phenolic compounds belonging to hydroxybenzoic acids and flavones subclasses were identified in lower percentages. Overall, all extracts were rich in caffeoylquinic acids, rutin, and 3-feruloylquinic acid (Figure 1; Table 2). In the case of *A. absinthium*, 3-feruloyl-4-caffeoylquinic acid and 3,5-diferuloylquinic acid were not found in the ethanol extract of *A. absinthium* flowers, and caffeoyl tartaric acid was not found in the ethanol extract of *A. absinthium* leaves.

### 2.3. In Vitro Antioxidant Activity

*A. absinthium* and *A. annua* flower and leaf ethanol extracts displayed in vitro antioxidant activities. The concentration of all extracts needed to inhibit 50% of DPPH radicals was above 50 μg TE/mL and lower than 200 μg TE/mL, indicating a moderate antioxidant capacity. The *A. absinthium* leaf extract had a weaker effect on the DPPH radical (*p* < 0.01). The H_2_O_2_ and FRAP scavenging activities were higher than those of Trolox (*p* < 0.001), with no significant differences between the extracts (*p* > 0.05). The NO scavenging activity was more robust than that of quercitin (*p* < 0.001), the *A. absinthium* leaf extract had the highest NO scavenging activity (*p* < 0.001) (Table 3).

### 2.4. In Vitro Antiproliferative Activity

The *A. absinthium* and *A. annua* flower and leaf ethanolic extracts display an important antiproliferative activity against the ovarian tumor cell lines A2780cis, OVCAR-3, and OAW-42 (Table 4), with all IC50 values standing under 60 μg/mL. When compared with the tumor cell populations, the antiproliferative activity against the normal HaCaT cells was lower in all cases, except in the case of the *A. absinthium* leaf extract on OVCAR-3 cells, indicating that they are selective to a certain degree. In further testing, the IC50 values were used to treat the cells and to elucidate features of the potential mechanisms of action of the *A. absinthium* and *A. annua* flower and leaf extracts.

**Figure 2:**
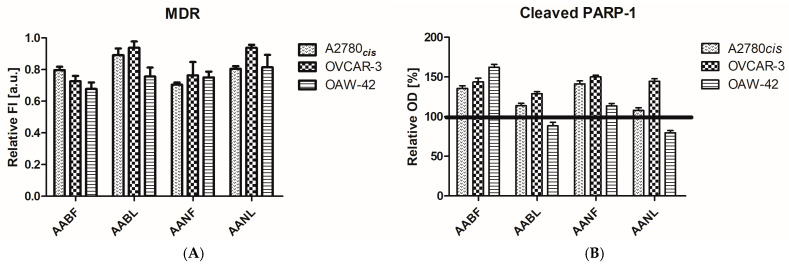
The in vitro effect of *A. absinthium* and *A. annua* flower and leaf ethanol extracts: (**A**) On the multidrug resistance (MDR) proteins secreted by the cells expressed as relative fluorescence intensity (FI). (**B**) On the relative optical density (OD) of the intracellular Asp214 fragment from the cleaved poly-ADP ribose polymerase-1 (PARP-1) enzyme in the human ovarian tumor cell lines A2780cis, OVCAR-3 and OAW-42 subjected to a 24 h treatment with the IC50 concentrations of the four extracts. The threshold line indicates the reference, untreated tumor cells’ PARP-1 levels. AABF—*A. absinthium* flowers; AABL—*A. absinthum* leaves; AANF—*A. annua* flowers; AANL—*A. annua* leaves.

The tumor cell lines A2780cis, OVCAR-3, and OAW-42 were subjected to 24 h treatment with the IC50 concentration of each extract (according to Table 4), and the modulation of the MDR transporter protein and the apoptosis-related, cleaved form of the PARP-1 enzyme was assessed (Figure 2) with fluorescence and immune-enzymatic methods accordingly, as described.

The fluorescence intensity of each treated tumor cell sample was normalized up to that of the untreated control cells in order to obtain relative MDR values. For all extracts, a decrease in MDR was consistently observed in the tumor cells (Figure 2A), especially for the activity of *A. annua* flowers on the A2780cis, OVCAR-3, and OAW-42 cell lines, the *A. absinthium* flower extract on the OVCAR-3 cell line, and the *A. absinthium* leaves and *A. annua* leaves on the OAW-42 cell line. In these cases, the decrease in the level of MDR proteins was statistically significant (one-way variance analysis, Bonferroni post-test in the 95% interval of confidence).

The expression of the cleaved form of PARP-1, the 89 kDa PARP-1 fragment, containing the Asp214 protein is a reliable biomarker of apoptotic cell death mechanisms. The cleaved PARP-1 displayed an increasing tendency in all treated cell lines, except the two leaf extracts in OAW-42 cells (Figure 2B), which are positioned under the 100% threshold corresponding to untreated cells. The *A. absinthium* flower extract triggered a statistically significant upregulation of apoptosis-linked PARP-1 cleavage in the A2780cis, OVCAR-3, and OAW-42 cell lines, being constantly active against all the analyzed ovarian cell lines, and, in A2780cis and OAW-42, it was the most efficient of all the extracts (one-way analysis of variance, Bonferroni post-test, in the 95% interval of confidence). The *A. annua* flower extract resulted in a notable increase in PARP-1 in the A2780cis and OVCAR-3 cells. The *A. absinthium* and the *A. annua* leaf extracts were efficient only in the OVCAR-3 cell line. Overall, the OVCAR-3 cell line was the most affected by the extracts, with the effect of the four extracts on PARP-1 in this cell line having almost the same scale, with only one notable difference (*A. absinthium* leaf extract vs. *A. annua* flower extract). In the OAW-42 cell line, PARP-1 regulation was divergent, with statistically significant differences observed between all extracts.

The PCA for AABF, AABL, AANF, and AANL showed that MDR and cleaved PARP-1 were positively correlated.

### 2.5. In Vivo Anti-Inflammatory Activity

Inflammation significantly increased the level of NfkB-p65, and the treatments with TX and DICLO lowered the level of this transcription factor (*p* < 0.001). Both the *A. absinthium* flower and leaf ethanol extracts reduced the level of NfkB-p65, but only the AABF 100%, AABF 50%, AABL 100%, and AABL 50% effects were significant (*p* < 0.05). The *A. absinthium* flower and leaf ethanol extracts’ effects on NfkB-p65 were weaker than those of TX and DICLO (*p* < 0.01). *A. annua* flower and leaf ethanol extracts had a low inhibitory activity on NfkB-p65 (*p* < 0.05), it was not concentration-dependent, and it was smaller than those of TX and DICLO (*p* < 0.001) too (Table 5).

IL-1 and IL-18 increased significantly in INFL animals *p* < 0.001), and all plant extracts in all dilutions lowered their concentrations significantly (*p* < 0.001). TX and DICLO lowered IL-1 concentration too (*p* < 0.001), and the effects were as strong as those of the plant extracts. On IL-18, TX had a lesser inhibitory effect (*p* < 0.01), and DICLO had no significant activity (*p* > 0.05) (Table 5).

GSDMD increased significantly in the INFL group (*p* < 0.001). TX had a small inhibitory effect (*p* < 0.05), and DICLO caused a stronger reduction (*p* < 0.01). For all tested plant extracts, the undiluted extracts had a significant inhibitory activity (*p* < 0.001) and the 50% concentrations had a lower inhibitory effect (*p* < 0.01). The flower extracts AABF 25% and AANF 25% did not affect GSDMD (*p* > 0.05), and the leaf extracts AABL 25% and AANL 25% had just a small amount of inhibitory activity (*p* < 0.05). The plant extract activity was better than that of TX and DICLO (Table 5).

The level of caspase-1 increased after inflammation induction *p* < 0.001), and TX and DICLO decreased it significantly (*p* < 0.001). *A. absinthium* flower and leaf ethanol extracts significantly lowered caspase-1 (*p* < 0.001) but with no significant differences between dilutions. The *A. annua* flower and leaf ethanol extracts reduced caspase-1 generation very significantly only in the AANF 100%, AANF 50%, AANL 100%, and AANL 50% animals (*p* < 0.001). AANF 25% and AANL 25% had a lesser inhibitory effect (*p* < 0.01). Only the undiluted plant extracts, respectively, AABF 100%, AABL 100%, AANF 100%, and AANL 100%, caused a greater reduction in the level of caspase-1 than TX and DICLO (*p* < 0.01) (Table 5).

### 2.6. In Vivo Antioxidant Activity

Inflammation caused a significant increase in the general oxidative stress biomarkers TOS and OSI (*p* < 0.001), and the treatments with the *A. absinthium* and *A. annua* flower and leaf ethanol extracts lowered TOS and OSI (*p* < 0.001) with no important differences between the type of extract or dilution of the extract. TX and DICLO had similar inhibitory activity on both TOS and OSI (*p* < 0.001), and the effects were as important as those of the extracts (*p* > 0.05). The general antioxidant biomarker TAC was lowered by INFL (*p* < 0.001), and the *A. absinthium* flower, *A. absinthium* leaf, *A. annua* flower, and *A. annua* leaf extract treatments increased TAC (*p* < 0.01). TX and DICLO improved TAC, too (*p* < 0.01), and the effects were smaller than those of the plant extracts (*p* < 0.01) (Table 6).

Inflammation caused a significant increase in protein oxidation, as measured by AOPP (*p* < 0.001), and TX and DICLO reduced AOPP importantly (*p* < 0.001). The ethanol extracts of *A. absinthium* flowers, *A. absinthium* leaves, *A. annua* flowers, and *A. annua* leaves induced a significant reduction in AOPP, too (*p* < 0.001), and, excepting AANF 25%, in all groups, the results were as good as for TX and DICLO (*p* > 0.05) (Table 6).

The lipid peroxidation evaluation with MDA showed a significant increase in the INFL group (*p* < 0.001), and TX (*p* < 0.001) and DICLO (*p* < 0.01) reduced the MDA concentration. All tested *A. absinthium* and *A. annua* extracts lowered MDA, too (*p* < 0.01), with no significant differences between the type or concentration of the extract, or when compared to TX and DICLO (*p* > 0.05) (Table 6).

Inflammation stimulated NO synthesis (*p* < 0.001), and the antioxidant substance TX and the anti-inflammatory drug DICLO reduced NO synthesis (*p* < 0.001) without reaching the CONTROL level. The AABF and AANL extracts caused a concentration-dependent reduction in NO, with the highest concentration having the best inhibitory activity (*p* < 0.001). AABL and AANF reduced NO synthesis too, but not in a concentration-dependent way (*p* < 0.01) (Table 6).

The content of 3-NT, as an RNS marker, increased during inflammation (*p* < 0.001). TX, as an antioxidant, and DICLO, as an anti-inflammatory drug, caused a very significant reduction in 3-NT (*p* < 0.001). The *A. absinthium* and *A. annua* extracts lowered 3-NT (*p* < 0.001) in a concentration-dependent way, but without a significant difference in the activities of the different dilutions (*p* > 0.05) (Table 6).

The content of 8-OhdG, a marker of DNA oxidation, was increased by inflammation (*p* < 0.001) and reduced by TX and DICLO (*p* < 0.001). The ethanol extracts of *A. absinthium* flowers, *A. absinthium* leaves, *A. annua* flowers, and *A. annua* leaves lowered the level of 8-OhdG (*p* < 0.001) in a concentration-dependent way, with the higher concentration having the best inhibitory activity. The plant extracts were better inhibitors of DNA oxidation than TX and DICLO (*p* < 0.01) (Table 6).

SHs, as markers of serum antioxidants, were reduced by inducing inflammation (*p* < 0.001). TX and DICLO increased the level of SH almost to the CONTROL level (*p* < 0.01). The AABF and AABL extracts increased SH above the CONTROL level, in fact, to almost double the CONTROL level (*p* < 0.001), and AANF and AANL increased SH until it reached the CONTROL level (*p* < 0.001) (Table 6).

### 2.7. Liver and Renal Toxicity Assessment

The liver tests AST and ALT were in normal ranges in the INFL and in all the treated groups (*p* > 0.05). The renal tests of creatinine and urea were increased after inflammation induction (*p* < 0.05). Only the TX, DICLO, and AANL treatments lowered the levels of serum creatinine and urea (*p* < 0.01) (Table 7).

### 2.8. Correlations Analysis

**Figure 3:**
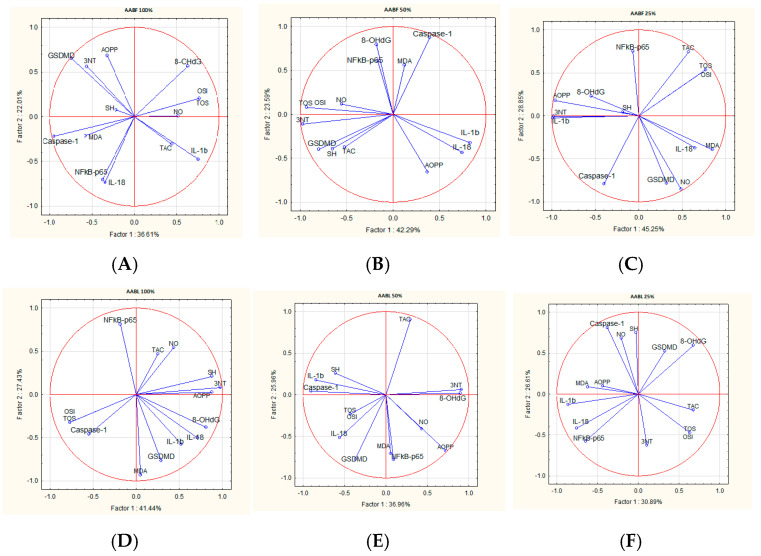
The PCA results of inflammatory and oxidative stress biomarkers based on the correlation matrix with PC1 and PC2 for *A. absinthium* flower and leaf ethanol extracts: (**A**) PCA for *A. absinthium* flowers 100%; (**B**) PCA for *A. absinthium* flowers 50%; (**C**) PCA for *A. absinthium* flowers 25%; (**D**) PCA for *A. absinthium* leaves 100%; (**E**) PCA for *A. absinthium* leaves 50%; (**F**) PCA for *A. absinthium* leaves 25%.

The treatments with *A. absinthium* and *A. annua* flower and leaf ethanol extracts decreased inflammation and OS markers, but these effects were differently correlated for each extract. The correlation analysis between inflammatory and OS parameters varied according to the plant extract type and concentration. The PCA analyzed parameter variability by the comparisons of the first principal component (PC1) and the second component (PC2) (Figure 2 and Figure 3).

In AABF 100% and AABF 50%, TOS and OSI were best correlated to 8-OhdG and NO, and NF-kB-p65 to IL-18 and caspase-1. In AABF 25%, there was a good positive correlation between TOS, OSI, MDA, and NF-kB-p65 and IL-1b. In AABL 100% and AABL 50%, TOS and OSI were best correlated to MDA, and, in AABL 25%, to NOx and 3NT. For the AABL dilutions, NF-kB-p65 was positively correlated with IL-1b and IL-18. (Figure 3).

**Figure 4:**
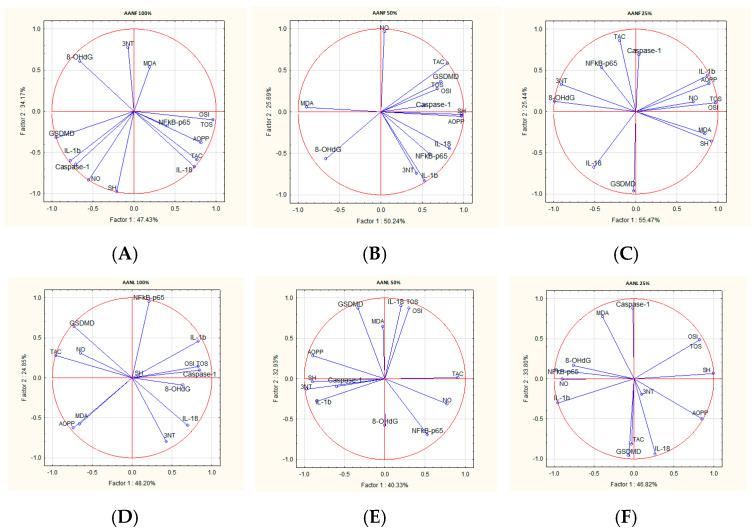
The PCA results of oxidative stress and inflammatory biomarkers based on the correlation matrix with PC1 and PC2 for *A. annua* flower and leaf ethanol extracts: (**A**) PCA for *A. annua* flowers 100%; (**B**) PCA for *A. annua* flowers 50%; (**C**) PCA for *A. annua* flowers 25%; (**D**) PCA for *A. annua* leaves 100%; (**E**) PCA for *A. annua* leaves 50%; (**F**) PCA for *A. annua* leaves 25%.

In AANF, TOS and OSI were positively correlated with AOPP. In AANF 100% and AANF 50%, NF-kB-p65 was positively correlated with IL-18, and, in AANF 25%, with caspase-1. In AANL 100%, TOS and OSI were positively correlated with 8-OhdG, in AANL 50%, with MDA, and, in AANL 25%, with AOPP. In AANL, NF-kB-p65 was positively correlated with IL-1b (Figure 4).

## 3. Discussion

The current study explored the phytochemical constituents, the in vitro antioxidant activity, the in vivo anti-inflammatory and antioxidant activity, and the antiproliferative potential of *A. absinthium* and *A. annua* flower and leaf ethanol extracts. The results revealed that these extracts have a high polyphenolic content, have an important anti-inflammatory and antioxidant potential, and can reduce cell proliferation.

In recent years, there has been a growing research interest in plant phenolics’ pharmacological properties, mainly due to their antioxidant activity [25]. When comparing with other *Artemisia* plants harvested from the same geographical region, AABF, AABL, and AANL had a lower TPC than *A. abrotanum* and *A. dracunculus,* and only the TPC of AANF was as important as that of *A. dracunculus* [24]. The TFC estimation in *A. absinthium* and *A. annua* flower and leaf ethanol extracts revealed the presence of flavonoids in lower concentrations than in *A. abrotanum* and *A. dracunculus* [24]. These variations regarding the total flavonoids and total phenolics of different Artemisia plant extracts are a consequence of genetic and environmental factors as well as the plant organs used for extract preparation [26].

The HPLC-DAD-ESI MS profile of *A. absinthium* and *A. annua* flower and leaf ethanol extracts detected 26 phenolic compounds from the phenolic acids group and flavonoids group, like caffeoylquinic acids, rutoside, and 3-feruloylquinic acid, which were previously identified in other *Artemisia* species too [24]. The results followed previously reported data on an *A. absinthium* extract which mentioned the presence of phenolic acids (e.g., gallic, coumaric, vanillic, syringic, and chlorogenic salicylic acids) and flavonoids (e.g., quercetin and rutin) [3]. Further, in the *A. absinthium* flower ethanol extract, 3-Feruloyl-4-caffeoylquinic acid and 3,5-Diferuloylquinic acid were not detected, and, in the *A. absinthium* leaf ethanol extract, caffeoyl tartaric acid was not detected.

For the *A. absinthium* and *A. annua* flower and leaf ethanol extracts’ antioxidant properties determinations, different antioxidant assays, like DPPH, FRAP, H_2_O_2_, and NO assays, were performed. The effect of antioxidants on DPPH radical scavenging is due to their hydrogen-donating ability. The DPPH radical scavenging activity for the *A. absinthium* and *A. annua* flower and leaf ethanol extracts was found to be smaller than that of TX, but significant. AABL was least efficient. The presence of important multifunctional phenols may have been responsible for the antioxidant activity due to their ability to donate electrons and neutralize the highly reactive DPPH radical [7,25,27]. The FRAP assay does not involve the scavenging of added radicals or radical generation. It uses a redox reaction in which an oxidant molecule changes color when ferric ions (Fe^3+^) are reduced to ferrous ions (Fe^2+^) [28]. The *A. absinthium* and *A. annua* flower and leaf ethanol extracts showed good reducing power. The H_2_O_2_ scavenging test demonstrated the ability of *A. absinthium* and *A. annua* flower and leaf ethanol extracts to diminish the stable radical. This may be attributed to the presence of phenolic compounds that can donate electrons to hydrogen peroxide with the formation of H_2_O [29]. Trolox had better in vitro antioxidant activity than *A. absinthium* and *A. annua* flower and leaf ethanol extracts when measured by DPPH, FRAP, and H_2_O_2_ assays.

Nitric oxide, an unstable radical, reacts with oxygen to produce the stable products nitrate and nitrite. In the NO scavenging assay, the antioxidant compounds from the *A. absinthium* and *A. annua* flower and leaf ethanol extracts competed with oxygen to react with NO and inhibit nitrite generation [30]. The plant extracts’ effects were smaller than the effect of quercetin, especially for AABL.

The *A. absinthium* and *A. annua* flower and leaf extracts displayed in vitro antiproliferative effects against human ovarian tumor cell populations, and a certain degree of selectivity (Figure 2). The A2780cis cell line, having a higher proliferation rate than OVCAR-3 or OAW-42, was proven to be the most sensitive to relatively short-term exposure (24 h) to the extracts. Two mechanistic features were analyzed: the capacity of the extracts to modulate the MDR1 multidrug resistance pumps and the apoptosis-related Asp214 cleaved PARP-1 protein. To evaluate the *Artemisia* varieties’ capacity to counteract drug resistance, which is a major challenge in ovarian cancer treatment, we selected as an in vitro biological model three platinum drug-resistant tumor cell populations. The cell lines expressed a significant basal MDR protein level, which was downregulated by the extracts following the in vitro treatment for 24 h. The cleaved PARP-1 protein fraction was detected in the untreated ovarian cell lysates, with the presence of the enzyme being a characteristic of tumor cells and a therapeutic target for PARP-1 inhibitors in ovarian cancer. The IC50 values correlate well with the percentage of the MDR1 inhibition (one-tailed Pearson correlation, *p*-value = 0.02), while no correspondence was found between the IC50 and PARP-1, and between MDR1 inhibition and PARP-1 cleavage, respectively. The *A. annua* flowers exerted their biological effect against the A2780cis, OVCAR-3, and OAW-42 cell lines by inhibiting the MDR1 drug transporter pumps, while the *A. absinthium* flower extracts triggered the PARP-1 enzyme cleavage consistently in all cell lines. The *A. absinthium* leaf and *A. annua* leaf extracts exhibited cytotoxicity against the tumor cells and they were able to modulate the molecular targets MDR1 and PARP-1. A stronger statistical significance was present for MDR1 in the OAW-42 cell line, and for PARP-1 in the OVCAR-3 cell line only, which denotes the fact that the leaf extracts induced different mechanisms and cell signaling alongside the two studied paths [31].

Based on the in vitro observations and considering that a major limitation of these tests is the non-physiological measurement conditions [28], we continued by performing in vivo tests to evaluate the anti-inflammatory and antioxidant activity.

The inflammatory response is a vital defense mechanism of the organism triggered by PAMPs or DAMPs that activate the immune system. Acute inflammation is associated with the release of pro-inflammatory mediators followed by vascular leakage and leukocyte recruitment and activation. In normal conditions, it is self-limited, and the resolution and recovery are associated with the induction of anti-inflammatory mechanisms, neutrophil apoptosis, removal of apoptotic cells and cellular debris, and restoration of vascular permeability.

NF-κB refers to a family of transcription factors that is crucial for regulating both normal physiological processes and pathological conditions. It exists as an inactive complex in the cytoplasm, and activation is initiated by external or internal stimuli (e.g., TNFα). Activation causes IκB kinase degradation, and the remaining NF-κB dimer (e.g., p65/p50 or p50/p50) translocates to the nucleus where it binds to a DNA consensus sequence of target genes. NF-κB subunits can also migrate into the mitochondrion, where they interact with mitochondrial mtDNA triggering the expression of some proteins or stimulating apoptosis [32]. There are two different NF-κB pathways, the canonical and non-canonical NF-κB pathways. Activation of the canonical NF-κB pathway causes a quick but transient transcriptional activity that regulates the expression of important proinflammatory mediators. The activation of the non-canonical NF-κB pathway is slow but sustained, playing a key role in the development of immune cells, lymphoid organs, and immune responses. When innate immune cells detect microbial PAMPs (pathogen-associated molecular patterns) or DAMPs (damage-associated molecular patterns) from injured tissues, the canonical NF-κB pathway is activated, leading to the production of proinflammatory cytokines, such as TNF-α and IL-1β, along with other inflammatory mediators [33]. These cytokines, in turn, further activate the canonical NF-κB pathway, creating a self-perpetuating inflammatory cycle. Dysregulated NF-κB activity can lead to inflammatory and autoimmune diseases, and even cancer [34]. The *A. absinthium* and *A. annua* flower and leaf ethanol extracts lowered NF-κB in a concentration-dependent way, with the highest dilution having the best effect. The effect on NF-κB was smaller than those of TX and DICLO.

The canonical NLRP3 inflammasome pathways can be also stimulated by PAMPs or DAMPs [35]. This process requires two independent stimuli. The first one, the priming step, occurs through transcriptional regulation: toll-like receptor (TLR) stimulation increases the expression of pro-IL-1b, pro-IL-18, and NLRP3 inflammasome proteins. The second one causes inflammasome formation and activation of caspase-1 autocleavage. Then, caspase-1 will activate proIL-1β to IL-1β and proIL-18 to IL-18, which, consequently, induce the expression of other pro-inflammatory cytokines, finally leading to a “storm” of cytokines. Caspase-1 will also activate gasdermin D (GSDMD), which then forms pores in the plasma and intracellular membranes, leading to cell pyroptosis [36]. The NLRP3 noncanonical inflammasome pathways activate caspase-4/11, caspase-5, or caspase-8, which may modulate cell death by apoptosis or necrosis [35].

While a balanced inflammatory response favors inflammation resolution and tissue healing, excessive NLRP3 activation causes detrimental effects and facilitates chronic inflammatory diseases, autoimmunity, and cancer development [37]. For all these reasons, the NLRP3 inflammasome has become a target for anti-inflammatory therapies.

Polyphenols have several pharmacological activities, such as anti-inflammatory, antioxidant, and anticancer effects, that have been scientifically documented [36]. Some flavonoids and polyphenols modulate immune function via stimulatory or inhibitory activity on the NLRP3 inflammasome [35]. Regarding flavonoids, it has been demonstrated that their anti-inflammatory activity consists of immune cell regulation and the suppression of pro-inflammatory transcription factors and inflammatory mediators, like cytokines and chemokines [38].

For the anti-inflammatory activity assessment, NF-kB-p65, IL-1b, IL-18, caspase-1, and GSDMD were measured to evaluate canonical NLRP3 inflammasome activation. The *A. absinthium* and *A. annua* flower and leaf ethanol extracts with rich polyphenol contents had anti-inflammatory effects on the canonical NLRP3 inflammasome pathway via reductions in IL-1β, IL-18, caspase-1, and GSDMD in turpentine oil-induced experimental inflammation. On IL-1β and IL-18, the plant extracts’ inhibitory activities were as good as those of the anti-inflammatory drug DICLO. Caspase-1 and GSDMD concentrations were reduced in a concentration-dependent way, with the lower dilution having the best effect. The *A. absinthium* and *A. annua* flower and leaf ethanol extracts’ inhibitory activities on the NLRP3 inflammasome were similar or better than those of TX and DICLO. These results point to the anti-inflammatory potential of *A. absinthium* and *A. annua* flower and leaf ethanol extracts via inhibition of the canonical NLRP3 inflammasome pathway.

In healthy conditions, there is an equilibrium between ROS formation and endogenous antioxidant defense mechanisms. Normal cellular metabolism produces most of the ROS, such as singlet oxygen and hydrogen peroxide, in cells through the mitochondrial respiratory chain. These ROS have vital roles in the activation of intra- and extracellular metabolic signaling pathways [39]. In hypoxic conditions, the mitochondrial respiratory chain generates nitric oxide (NO), which reacts with ROS and produces reactive nitrogen species (RNS). In inflammation, leukocytes present in the injured tissue enhance the production and release of ROS and RNS. Further, ROS and RNS react with other molecules, like proteins, lipids, and nucleic acids, and can produce other, additional reactive species [39].

During acute inflammation, excessive tissue injury and neutrophil activation are associated with excessive ROS and RNS production, leading to inflammation-dependent OS [16,18,40]. Moreover, OS increases NF-κB expression and amplifies the inflammatory response [41]. In this way, such an inflammatory-dependent OS environment can harm healthy neighboring cells, and, after a long period, may even trigger carcinogenesis [39].

The antioxidant activities of natural plant compounds have been frequently reported. Plants’ natural antioxidants protect against the generation of free radicals and, therefore, are one of the more valuable therapeutic agents to reduce the development and progression of diseases triggered by OS [39]. Under normal conditions, flavonoids have a direct antioxidant activity by scavenging ROS and chelating metal ions, and an indirect antioxidant activity by stimulating the synthesis and activation of antioxidant enzymes and by suppressing pro-oxidant enzymes [38].

The oxidative status of the rats from all the groups was determined using the following OS biomarkers: TOS, TAC, OSI, AOPP, MDA, NO, 3-NT, 8-OHdG, and SH [42]. TOS measured the total pro-oxidant state, TAC evaluated the total antioxidant state, and OSI measured the severity of OS [10]. The TOS and OSI concentrations increased and TAC was reduced in the serum of rats with turpentine oil-induced inflammation. Like in previous studies, AABF, AABL, and AANL plant extracts had effects comparable with TX and DICLO on TOS and OSI, and only AANF had a lower level of inhibitory activity. On TAC plant extracts, the effects were better than those of the antioxidant TX and anti-inflammatory drug DICLO. Their results confirmed the in vivo antioxidant potential of these *Artemisia* extracts.

Further, by analyzing specific oxidized molecules, respectively, AOPP, MDA, NO, 3-NT, and 8-OHdG, we found increased serum concentrations in rats with turpentine oil-induced inflammation. AOPPs are markers of plasma protein oxidative damage. The *A. absinthium* and *A. annua* flower and leaf ethanol extracts reduced AOPP. This effect was as important as those of TX and DICLO for AABF, AABL, and AANL, with only AANF having a lesser inhibitory activity. These results are significant because AOPP amplifies the inflammatory response and OS via activation of the NF-kB-dependent pathway, and ROS production increases [40]. MDAs are secondary metabolites of lipoperoxides resulting from lipid oxidative damage. The *A. absinthium* and *A. annua* flower and leaf ethanol extracts reduced MDA as much as TX and DICLO. MDA reduction is an important effect because these molecules are diffusible products that can create a vicious cycle by extending the inflammatory response like AOPP does [43]. Moreover, lipid peroxidation may activate apoptosis in all cells [42].

The inflammatory mediator upregulated the expression of inducible nitric oxide synthase (iNOS) in activated phagocytes, causing the synthesis and secretion of large amounts of NO, an important cytotoxic/cytostatic mediator of nonspecific immunity. In mammals, there are three more NOS isoforms, respectively, endothelial NOS (eNOS), neuronal NOS (nNOS), and mitochondrial NOS (mtNOS). These NOS produce only small amounts of NO, with no toxic effects [44]. NO concentrations were measured indirectly with the Griess assay. Regarding *A. absinthium herba* extracts, it was previously demonstrated that they exhibit anti-inflammatory activity through inhibition of the expression of some proinflammatory mediators, such as NF-*κ*B, tumor necrosis factor-*α* (TNF-*α*), inducible nitric oxide synthase (iNOS), prostaglandin E-2 (PGE2), and cyclooxygenase-2 (COX-2). Some studies reported that the antioxidant activity of *A. absinthium* was linked to its phenolic compounds (gallic acid, coumaric acid, vanillic acid, syringic acid, and chlorogenic salicylic acid) and flavonoids (quercetin and rutin) [3]. Extracts of *A. annua herba* have anti-inflammatory and antioxidant activities via the repression of NF-kB, MAPK pathway activation, and inhibition of TNF-α, IL-6, and iNOS gene expression [39]. In the present study, we found that *A. absinthium* and *A. annua* flower and leaf ethanol extracts reduced NO production in a concentration-dependent way, with the higher concentration having the best inhibitory activity. Only AANF was as efficient as TX and DICLO in all dilutions. Excesses of both ROS and NO increased RNS formation in inflammation animals. Therefore, peroxynitrite (ONOO-), an RNS that causes protein tyrosine nitration resulting in 3-nitrotyrosine (3NT) [43], was reduced by *A. absinthium* and *A. annua* flower and leaf ethanol extracts too.

The compound 8-OHdG is a reliable biomarker that measures oxidative DNA damage, genetic instability, epigenetic changes, and even mutations [45]. *A. absinthium* and *A. annua* flower and leaf ethanol extracts reduced DNA oxidation significantly, but the effects were smaller than those of TX and DICLO. From the plasma antioxidants, SH was measured because it is the second biggest component contributing to TAC [44]. SH is a measure of sulfhydryl-containing organic compounds, and turpentine oil-induced inflammation reduced SH. *A. absinthium* and *A. annua* flower and leaf ethanol extracts had significant antioxidant activity by increasing SH concentration. Only AANF increased SH to the level of the CONTROL, and the rest of the extracts increased SH above the CONTROL level. It remains to be found if this effect is due to the stimulation of endogenous antioxidant synthesis due to some exogenous sources from the plant extracts.

Flavonoids are important natural phenols synthesized in plants as bioactive secondary metabolites responsible for their pharmacological activities. There is accumulating evidence that many flavonoids exert antioxidant, anti-inflammatory, immunomodulatory, and anticancer activities.

Furthermore, by acting as pro-oxidants, arresting the cell cycle, inducing cancer cell death by apoptosis or autophagy, and suppressing cancer cell proliferation and invasiveness, flavonoids exert anticancer activity [38]. Chronic inflammation is an important risk factor for tumor development; therefore, anti-inflammatory effects are important in promoting the antitumor activity of immune cells. Flavonoids exert a wide range of anticancer effects and, therefore, could serve as potential compounds for further studies on the development of novel cancer chemopreventive agents [38].

Although *Artemisia* spp. and its constituents show great potential as functional foods and safe complementary medicines, some adverse neurotoxic effects have been described. *A. absinthium* is accepted nowadays in foods and alcoholic beverages, but, according to the European Food Safety Authority (EFSA), the content of thujone from *Artemisia* must not exceed 10 mg/kg, and, according to the European Medicines Agency (EMA), dosage should not be more than 3 mg/day/person. *A. annua* is considered safe and nontoxic up to 5000 mg/kg of the extract [3]

The *A. absinthium* extracts improved hepatic function, resulting in lower serum aspartate (AST) and alanine aminotransferase (ALT) activity. The proposed hepatoprotective mechanisms include liver microsomal drug-metabolizing enzyme suppression, free radical scavenging activity, and calcium channel blockage [3]. In the present study, inflammation induction did not increase AST and ALT above normal Wistar rat values [46], and the *A*. *absinthium* and *A. annua* flower and leaf ethanol extracts had no hepatotoxic effects.

The kidney has many resident immune cells that, once activated, produce inflammatory mediators that can trigger kidney injury [47]. At the same time, the kidney is highly susceptible to injury from proinflammatory cytokines and oxidative stress because the kidneys do not have anti-inflammatory and antioxidant defenses [48]. These mechanisms explain the creatinine (normal value 0.2–0.7 mg/dL) and urea (normal value 28.43–39.15 mg/dL) increase after inflammation induction in rats [46]. *A. absinthium* and *A. annua* flower and leaf ethanol extract treatments lowered creatinine and urea, but only AANL brought them to the normal level, as did TX and DICLO.

## 4. Materials and Methods

### 4.1. Chemicals

Acetonitrile(CH_3_CN), ethanol(C_2_H_5_OH), methanol (CH_3_OH), hydrochloric acid (HCl), ammonium iron (II) sulfate ((NH_4_)_2_Fe(SO_4_)_2_·6H_2_O), diethylether, vanadium (III) chloride (VCl_3_), sulfanilamide (C_6_H_8_N_2_O_2_S),N-(1-Naphthyl)ethylenediamine dihydrochloride (C_12_H_14_N_2_)], xylenol orange [o-cresosulfonphthalein-3,3-bis (sodiummethyliminodiacetate), acetic acid, sulfuric acid, hydrochloric acid, glacial acetic acid, glycerol,ortho-dianisidine dihydrochloride (3-3′-dimethoxybenzidine), thiobarbituric acid, o-Phthalaldehydealuminum chloride-1-Ethyl-3-methylimidazolium chloride, hydrogen peroxide (H_2_O_2_), sodium hydroxide, sodium carbonate, sodium nitrite, sodium nitroprusside, Folin-Ciocalteu′s phenol reagent, potassium iodide, chloramine-T, thiobarbituric acid trichloroacetic acid, and 5,5′-Dithio-bis-(2-nitrobenzoic acid) were obtained from Merck (Darmstadt, Germany); luteolin, gallic acid, rutin, quercetin, and chlorogenic acid analytical standards were provided Sigma (St. Louis, MO, USA); trolox (6-hydroxy-2,5,7,8-tetramethylchroman-2-carboxylic acid) was bought from Alfa-Aesar (Karlsruhe, Germany); rat’s ELISA kits were bought from Elabscience Bionovation Inc. (Houston, TX, USA) and MyBiosource (San Diego, CA, USA); reagents for testing AST and ALT (aminotransferases), as well as creatinine and urea, were obtained from BioSystems Diagnostic (Ilfov, Romania). All chemicals used were of ultrapure grade, and type I reagent grade deionized water was employed in the procedures. For HPLC analysis, ultrapure water was obtained with the Millipore Direct-Q UV system (USA).

### 4.2. Plant Material and Plant Extract Preparation

*A. absinthium* (Voucher 666161) and *A. annua* (Voucher 670071) flowers and leaves were obtained in June 2021 from the Botanical Garden “Alexandru Borza” in Cluj-Napoca (46°45′36″ N and 23°35′13″ E) and were taxonomically identified at Babes-Bolyai University of Cluj-Napoca. Fresh *A. absinthium* and *A. annua* flowers and leaves (fragments of 0.5–1 cm) were extracted following the Squibb cold re-percolation method [9]. The extraction was performed in 70% ethanol for 3 days at room temperature. The obtained extracts were as follows: *A. absinthium* flowers (1:1 g/mL (*w*:*v*)), *A. absinthium* leaves (1:1.2 g/mL (*w*:*v*)), *A. annua* flowers (1:1.2 g/mL (*w*:*v*)), and *A. annua* leaves (1:1.2 g/mL (*w*:*v*)).

### 4.3. Quantitative Phytochemical Analysis

*A. absinthium* flower and leaves and *A. annua* flower and leaf extracts were characterized for their total polyphenol content (TPC) and total flavonoid content (TFC) as previously described [49,50]. The TPC values were expressed as milligrams of gallic acid equivalents per gram of dry-weight plant material (mg GAE/g d.w. plant material), while TFC was reported as milligrams of quercetin equivalents per 100 g of dry-weight plant material (mg QE/100 g d.w. plant material).

### 4.4. Qualitative Phytochemical Analysis

High-performance liquid chromatography (HPLC) (Agilent 1200) with diode array detection (DAD) was coupled with a single quadrupole mass spectrometer (Agilent 6110 MS) to analyze *A. absinthium* and *A. annua* flower and leaf extracts. Separation was performed on an XDB C18 Eclipse column using gradient elution with acetic acid/acetonitrile–water mixtures as previously described Phenolic compounds were detected at 280 nm (phenolic acids) and 340 nm (flavonoids), and further identified based on UV spectra, mass spectra, and retention times. Quantification was performed using standard calibration curves of chlorogenic acid (R^2^ = 0.9937, LOD = 0.41 μg/mL, LOQ = 1.64 μg/mL), luteolin (R^2^ = 0.9972, LOD = 0.26 μg/mL, LOQ = 0.95 μg/mL), and rutin (R^2^ = 0.9981, LOD = 0.21 μg/mL, LOQ = 0.84 μg/mL). Data analysis was conducted with Agilent ChemStation software (B.04.03) [51].

### 4.5. In Vitro Antioxidant Activity Analysis

#### 4.5.1. 2,2-Diphenyl-1-picrylhydrazyl (DPPH) Radical Scavenging Capacity

The DPPH radical scavenging capacity of *A. absinthium* and *A. annua* flower and leaf extracts was evaluated as previously described [49,50]. Extracts (3 mL) were mixed with 1 mL of 0.1 mM DPPH methanol solution and incubated in the dark for 30 min at room temperature. Absorbance was measured at 517 nm, and scavenging activity (AA%) was calculated using the next formula: AA% = [(A control − A sample)/A control] × 100. Results were expressed as IC_50_ (μg TE/mL), with thresholds for strong (≤50 μg/mL), moderate (50–100 μg/mL), and negligible (≥200 μg/mL) antioxidant activity [10].

#### 4.5.2. Ferric Reducing Antioxidant Power (FRAP) Assay

The reduction capacity of the extracts was assessed using the FRAP assay [11,28]. The extracts (100 μL) were mixed with 3.4 μL of FRAP reagent, incubated for 30 min, and absorbance was measured at 593 nm. The results were expressed as IC_50_ (μg TE/g d.w.).

#### 4.5.3. Hydrogen Peroxide (H_2_O_2_) Scavenging Activity

The ability of *A. absinthium* and *A. annua* flower and leaf ethanol extracts to scavenge hydrogen peroxide (H_2_O_2_) was determined as previously described [10]. The extracts were mixed with an H_2_O_2_ solution and incubated for 10 min, followed by absorbance measurement at 230 nm. The scavenging percentage was calculated using the next formula H_2_O_2_ scavenged %= (A control − A sample/A control) × 100, and results were expressed as IC_50_ (mg TE/g d.w.).

#### 4.5.4. Nitric Oxide (NO) Radical Scavenging Assay

Nitric oxide scavenging was evaluated as described previously [10], using sodium nitroprusside (SNP) and the Griess reagent. Extracts (0.5 mL) were incubated with 2 mL SNP solution (obtained from 2 mL SNP and 0.5 mL PBS, pH 7.4) for 2.5 h at 25 °C, followed by the reaction with sulphanilic (1 mL) acid and naphthyl ethylenediamine-dihydrochloride (1 mL). Absorbance was measured at 546 nm, and the percentage of inhibition was calculated using the following formula: NO scavenged % = (A blank − A sample/A blank) × 100. The results were expressed as IC_50_ (mg QE/g d.w.).

All in vitro antioxidant assays were conducted in triplicate, and measurements were taken using a UV–Vis spectrophotometer (Jasco V-350, Jasco International Co., Ltd., Tokyo, Japan).

### 4.6. In Vitro Antiproliferative Activity

The in vitro testing was performed on OVCAR-3 high-grade ovarian serous adenocarcinoma; OAW-42 ovarian cystadenocarcinoma, and HaCaT normal immortalized keratinocyte cell lines). A2780 cells were purchased from the European Collection of Authenticated Cell Cultures (Salisbury, UK), while OVCAR-3, OAW-42, and HaCaT were purchased from Cell Lines Service GmbH (Eppelheim, Germany). A2780 and OVCAR-3 cells were cultivated in RPMI-1640 media, supplemented with 10% and 20% fetal calf serum (FCS), respectively, and human insulin supplementation for OVCAR-3. OAW-42 and HaCaT cells were cultivated in high-glucose (4.5 g/L) Dulbecco’s Modified Eagles’ Medium media with 2 mM glutamine and 10% FCS. The cells were cultivated under standard conditions (at 37 °C, 5% CO_2_, in the dark, using a Galaxy 48RCO2 incubator (Eppendorf AG, Hamburg, Germany)). Subcultures were initiated in subconfluency, and viability testing was performed using 1% Trypan Blue staining, and measurement with the automatic cell counter EVE (NanoEnTek, Seoul, Republic of Korea) Passing was performed using a trypsin–EDTA solution.

#### 4.6.1. MTT Cytotoxicity Assay

The capacity of the four extracts to inhibit the growth of the cells was evaluated by the 3-(4,5-dimethylthiazol-2-yl)-2,5-diphenyltetrazolium bromide or MTT cytotoxicity test. Briefly, in a 96-well plate, the cells at a concentration of 10^5^ cells/mL cell culture media were incubated for 24 h. Plant extract stock solutions (750 μg dry-weight plant material /mL phosphate-buffered saline, PBS) were serial diluted (500, 250, 125, 62.5, and 31.25 μg/mL) and added to the cell culture to obtain final concentrations of 37.50, 25.00, 12.50, 6.25, 3.13, and 1.56 μg/mL. Every experiment was repeated three times across three independent passages. The half-maximal inhibitory concentration (IC50) values indicated the concentration of extracts needed to inhibit 50% of the cell [31].

#### 4.6.2. PARP-1 Cleavage ELISA Assay

The expression of the cleaved poly-ADP ribose polymerase-1 (PARP-1) enzyme was assessed with an ELISA assay kit (Abcam, Cambridge, United Kingdom A2780, OVCAR-3, and OAW-42 cells were cultured at 10^6^ cells/mL and treated with plant extracts, each at the IC_50_ concentration for 24 h. Untreated wells were used as a control. For every extract, three samples were processed from three distinct plates. Cells were harvested, rinsed with PBS, counted, and lysed in the extraction buffer. Lysates (50 μL) were incubated in PARp-antibody-coated 96 well plates on an orbital shaker (PSU-10i from BioSan, Riga, Latvia) at 300 rpm at room temperature for 2 h. Following washing, 50 μL detection antibody was added, incubated for 1 h, then treated with 50 μLof 3,3′,5,5′-tetramethylbenzidine (TMB) development solution and stopped with 1 N hydrochloric acid. The absorbance of samples was measured at 450/540 nm using a Sunrise ELISA reader, (Tecan Group, Männedorf, Switzerland). Relative concentrations were calculated using Magellan software (Infinite F50) [31].

#### 4.6.3. Multidrug Resistance (MDR) Assay

The ability of *Artemisia* extracts to counteract drug resistance was evaluated using a fluorimetric MDR Assay Kit (Abcam, Cambridge, UK). A2780, OVCAR-3, and OAW-42 cells were used at a concentration of 4 × 10^4^ cells/100 μL in 96-well black-wall plates (Costar, Corning, NY, USA) and were incubated for 24 h. Afterward, cells were treated with *A. absintium* or *A. annua* flowers and leaf extracts at the IC50 concentration, incubated for 24 h, treated with 10 μL MDR sensor solution, and incubated (1 h at 37 °C, 5% CO_2_). Following this, 100 μL/well MDR dye-loading solution was added, and the plates were incubated for another hour. Fluorescence intensity was measured at 490 nm excitation and 525 nm emission wavelengths using a Synergy LX microplate reader (BioTek Company, Winooski, VT, USA). Blank wells contained only cell culture media and reference wells contained untreated cells; the experiments were conducted in triplicate. The fluorescence intensity values of the blank wells were subtracted, and the fluorescence intensity yielded by the treated cells was related to the untreated control.

### 4.7. In Vivo Experimental Design

#### 4.7.1. Animal Subjects

Rats (adult males, albino Wistar rats) with weights between 200 and 250 g were taken from the Establishment for Breeding and Use of Laboratory Animals at the “Iuliu Hațieganu” University of Medicine and Pharmacy in Cluj-Napoca, Romania. Following the regulated laboratory conditions, the rats were kept in normal polypropylene cages. A standard 12 h light/dark cycle, a temperature of 25 ± 1 °C, and a relative humidity of 55 ± 5% were ensured throughout the experiment. The animals had free access to water and a standard granular diet. All procedures complied with Romanian national regulation 43/2014, which addresses the protection of animals employed in scientific research, and Directive 2010/63/EU. The Veterinary Sanitary Direction and Food Safety Cluj-Napoca approved this study (Approval No. 303/04.04.2022). The experiments were performed in triplicate.

#### 4.7.2. Experimental Protocol

The animals were randomly assigned to 16 groups (*n* = 9). Starting from day one, inflammation was induced in all groups, except the negative control (CONTROL), by intramuscular injection of turpentine oil (6 mL/kg body weight). Simultaneously, rats received daily oral treatments via gavage for 10 days as follows: the CONTROL and inflammation group (INFL) were given tap water (1 mL/rat/day); *Artemisia absinthium* flower extract (AABF) groups received three concentrations (100%, 50%, and 25%) (AABF 100%, AABF 50%, AABF 25%) at 1 mL/rat/day; *Artemisia absinthium* leaf extract (AABL) groups were treated with the same dilutions (AABL 100%, AABL 50%, AABL 25%) at 1 mL/rat/day; *Artemisia annua* flower extract (AANF) groups received the corresponding concentrations (AANF 100%, AANF 50%, AANF 25%) at 1 mL/rat/day; *Artemisia annua* leaf extract (AANL) groups were also administered three dilutions (AANL 100%, AANL 50%, AANL 25%) at 1 mL/rat/day. Additionally, an antioxidant control group was treated with Trolox (50 mg/kg body weight/day) (TX), and an anti-inflammatory drug group received diclofenac (10 mg/kg b.w./day) (DICLO). On the 11th day, animals were anesthetized with ketamine (60 mg/kg b.w.) and xylazine (15 mg/kg b.w.) [52], after which blood was collected via retro-orbital puncture. The serum was then separated and stored at −80 °C for further analysis.

#### 4.7.3. Oxidative Stress Markers Assessment

##### Total Oxidative Status (TOS)

Total Oxidative Status (TOS) was assessed by evaluating the oxidation of ferrous ions to ferric ions in the presence of reactive oxygen species (ROS) under acidic conditions. The findings were reported in μmol H_2_O_2_ equivalents per liter (μmol H_2_O_2_E/L) [53].

##### Total Antioxidant Capacity (TAC)

Total Antioxidant Capacity (TAC) was determined by measuring the ability of serum antioxidants to inhibit hydroxyl radical production through the Fenton reaction. The results were expressed in mmol Trolox equivalents per liter (mmol TE/L) [54].

##### Oxidative Stress Index (OSI)

The Oxidative Stress Index (OSI) was obtained after calculating the ratio between TOS and TAC and gives information on the degree of oxidative stress. The following formula was used: OSI (Arbitrary Unit) = TOS (mol H_2_O_2_ Equiv/L)/TAC (mmol Trolox Equiv/L).

##### 8-Hydroxydeoxyguanosine (8-OHdG)

The level of 8-hydroxydeoxyguanosine (8-OHdG), an indicator of oxidative DNA damage, was determined using an ELISA kit (E-EL-0028) following the manufacturer’s instructions. The results were recorded in ng/mL.

##### Advanced Oxidation Protein Products (AOPP)

Advanced Oxidation Protein Products (AOPPs), a marker of protein oxidation, were quantified through a spectrophotometric assay [55]. Samples and a chloramine-T blank were diluted to 10% in PBS, followed by the addition of potassium iodide and glacial acetic acid. The absorbance was then measured at 340 nm after the reaction, with the blank’s optical density subtracted before analysis. The results were expressed in µmol chloramine-T equivalents per liter (µmol chloramine E/L).

##### The Malondialdehyde (MDA)

Malondialdehyde (MDA), an indicator of lipid peroxidation, was measured using the thiobarbituric acid (TBA) method. Briefly, 0.1 mL of serum was mixed with 40% trichloroacetic acid (0.1 mL), followed by the addition of 0.67% TBA (0.2 mL). The mixture was heated in a boiling water bath (30 min) and then rapidly cooled in an ice bath. After centrifugation (3461× *g*, 5 min), the absorbance of the supernatant was recorded at 532 nm. The concentration of MDA in serum was expressed in nmol/mL [37].

##### Nitric Oxide Synthesis (NO)

Nitric oxide (NO) production was indirectly estimated by quantifying total nitrites and nitrates using the Griess reaction. Serum proteins were removed via extraction with a methanol/diethyl ether solution (3:1, *v*/*v*). Nitrates were reduced to nitrites with vanadium (III) chloride before adding the Griess reagent. Absorbance was measured at 540 nm, and results were reported in μmol nitrite per liter (μmol/L) [56,57].

##### 3-Nitrotyrosine (3NT)

The level of 3-Nitrotyrosine (3NT), a marker of oxidative damage caused by peroxynitrite [58], was measured using an ELISA kit (E-EL-0040) following the manufacturer’s protocol. The concentrations were reported in ng/mL.

##### Total Thiols (SHs)

Total thiols (SHs), an antioxidant marker, were quantified using Ellman’s reagent [59].

#### 4.7.4. Inflammatory Markers Assessment

The assessment of anti-inflammatory activity was conducted by measuring serum levels of nuclear factor Kappa B p65 (NfkB-p65) (E-EL-RO674), interleukin-1 beta (IL-1β) (E-EL-0012), interleukin-18 (IL-18) (E-EL-R0567), caspase-1 (ER0800), and gasdermin D (GSDMD) (MBS2705517) using ELISA kits according to the manufacturer’s guidelines. The concentrations of NfkB-p65 and GSDMD were expressed in ng/mL, whereas IL-1β, IL-18, and caspase-1 levels were reported in pg/mL.

#### 4.7.5. Toxicity Assessment

Hepatic toxicity was evaluated by measuring alanine transaminase (ALT) and aspartate aminotransferase (AST) levels, while renal toxicity was assessed by determining urea and creatinine concentrations. Spectroscopic analyses were performed using a UV–Vis spectrophotometer (Jasco V-350, Jasco International Co., Ltd., Tokyo, Japan). ELISA assays were carried out with a Biotek Microplate 50 TS washer and an 800 TS ELISA microplate reader (Agilent Technologies Inc., Santa Clara, CA, USA).

### 4.8. Statistical Analysis

The data are expressed as mean ± standard deviation (SD) for variables following a normal distribution. Group comparisons were performed using a one-way analysis of variance (ANOVA), followed by the Bonferroni–Holm post hoc test. Pearson’s correlation test and principal component analysis (PCA) were used for correlation analysis. A *p*-value below 0.05 was considered statistically significant. Statistical analyses were carried out using SPSS Statistics Version 26.0 for Windows (SPSS, Chicago, IL, USA) and GraphPad Prism Version 8.0 (GraphPad Software, San Diego, CA, USA).

## 5. Conclusions

In conclusion, the results of the present study provided sound scientific proof that *A. absinthium* and *A. annua* flower and leaf ethanol extracts possess high anti-inflammatory, antioxidant, and antiproliferative potential. Additionally, further phytochemical analyses and in vivo pharmacological studies are needed to explore the importance of *A. absinthium* and *A. annua* flower and leaf ethanol extracts as complementary medicinal treatments. Our results could be used to design and develop remedies for inflammatory diseases, inflammation-induced oxidative stress, and cancers.

## Figures and Tables

**Table 1:** Total polyphenols content and total flavonoid content of *A. absinthium* and *A. annua* flower and leaf ethanol extracts.

	Total Polyphenols Content mgGAE/g d.w. Plant Material	Total Flavonoid Content mg QE/100 g d.w. Plant Material
*A. absinthium* flowers (1 gr/1 mL)	1.67 ± 0.08	139.94 ± 10.87
*A. absinthium* leaves (1 gr/1.2 mL)	1.49 ± 0.02	84.94 ± 6.92
*A. annua* flowers (1 gr/1.2 mL)	2.16 ± 0.04	214.23 ± 32.07
*A. annua* leaves (1 gr/1.2 mL)	1.24 ± 0.01	120.21 ± 9.45

Note: values are expressed as mean ± SD (*n* = 3).

**Table 2:** Tentative identification of phenolic compound by HPLC-MS from *A. absinthium* and *A. annua* flower and leaf ethanol extracts.

Peak No.	R_t_ (min)	Compound	Concentration (μg/g)			
			*A. absinthium* Flowers	*A. absinthium* Leaves	*A. annua* Flowers	*A. annua* Leaves
1	3.22	3,5-Dihydroxybenzoic acid	49.91 ± 2.83	77.87 ± 4.24	26.43 ± 1.41	20.24 ± 1.50
2	11.04	3-Caffeoylquinic acid (Neochlorogenic acid)	185.42 ± 5.66	236.10 ± 11.24	55.45 ± 3.54	57.90 ± 3.50
3	12.57	5-Caffeoylquinic acid (Chlorogenic acid)	2659.23 ± 88.39	2823.06 ± 79.90	2232.88 ± 89.8	1064.44 ± 63.50
4	12.95	4-Caffeoylquinic acid (Criptochlorogenic acid)	88.67 ± 4.24	182.43 ± 6.29	46.31 ± 1.63	55.72 ± 2.25
5	13.21	Caffeoyl acid-glucoside	44.13 ± 1.34	191.64 ± 6.72	96.58 ± 3.54	82.71 ± 4.80
6	13.72	Iso-Ferulic acid	76.89 ± 5.66	130.95 ± 4.17	246.52 ± 15.4	110.12 ± 3.95
7	14.24	Caffeoyl tartaric acid	71.71 ± 5.02	n.d.	116.55 ± 4.95	85.84 ± 5.48
8	14.64	Apigenin-arabinosyl-glucoside	13.72 ± 1.34	13.97 ± 1.27	168.29 ± 7.07	118.22 ± 5.00
9	15.02	3-Feruloylquinic acid	166.47 ± 4.95	163.05 ± 3.54	1201.73 ± 70.71	546.98 ± 32.50
10	15.47	4-Feruloylquinic acid	64.54 ± 4.23	67.29 ± 4.95	98.49 ± 2.83	70.06 ± 6.50
11	15.88	5-Feruloylquinic acid	126.35 ± 9.90	175.81 ± 5.66	155.29 ± 16.26	109.01 ± 3.45
12	16.03	Quercetin-rutinoside (Rutin)	149.05 ± 11.31	220.72 ± 11.31	187.90 ± 12.02	89.04 ± 6.55
13	16.66	Apigenin-glucosyl-arabinoside	30.54 ± 2.12	83.69 ± 4.24	297.29 ± 7.78	113.69 ± 6.50
14	17.13	Isorhamnetin-rutinoside	234.74 ± 17.68	102.70 ± 4.95	429.73 ± 19.80	790.47 ± 13.45
15	17.52	3,4-Dicaffeoylquinic acid	467.33 ± 15.56	344.13 ± 11.31	1652.25 ± 67.18	1302.00 ± 80.50
16	17.86	3,5-Dicaffeoylquinic acid	1450.86 ± 74.25	1097.25 ± 62.93	1006.37 ± 48.79	805.10 ± 21.50
17	18.11	Quinic acid derivative	115.28 ± 5.66	141.81 ± 5.66	485.51 ± 6.36	149.02 ± 12.50
18	18.35	4,5-Dicaffeoylquinic acid	512.49 ± 21.21	458.49 ± 19.09	425.25 ± 16.26	369.09 ± 8.50
19	18.73	3-Feruloyl-4-caffeoylquinic acid	n.d	187.16 ± 12.02	107.04 ± 6.36	122.08 ± 6.50
20	19.42	4-Feruloyl-5-caffeoylquinic acid	125.82 ± 4.24	130.41 ± 4.24	1461.61 ± 60.81	794.42 ± 29.50
21	19.84	3,4-Diferuloylquinic acid	193.70 ± 5.66	152.57 ± 5.66	284.51 ± 13.44	252.47 ± 25.75
22	20.44	3,5-Diferuloylquinic acid	n.d	59.45 ± 6.36	170.22 ± 3.54	126.01 ± 8.50
23	21.32	5-Caffeoyl-4-feruloyl-quinic acid	117.41 ± 4.95	93.11 ± 4.24	436.15 ± 17.68	218.89 ± 17.65
24	22.91	3,4,5-Tricaffeoylquinic acid	498.85 ± 22.63	259.34 ± 5.66	233.76 ± 20.51	292.95 ± 14.00
25	23.68	3,5-Dihydroxy-6,7,4′-trimethoxyflavone	41.17 ± 3.54	63.02 ± 2.83	117.02 ± 7.78	71.59 ± 5.50
26	24.39	3,5-Dihydroxy-6,7,3′,4′-tetramethoxyflavone	113.29 ± 4.24	20.33 ± 3.54	108.81 ± 2.83	82.77 ± 9.50
		Total Phenolics	7597.65 ± 354.26	7476.46 ± 361.33	11,848.05 ± 502.05	7900.94 ± 428.13

Note: Values are expressed as mean ± SD (*n* = 3). n.d.—not detected

**Table 3:** In vitro antioxidant activity of the *A. absinthium* and *A. annua* flower and leaf ethanol extracts.

	DPPH μg TE/g d.w. Plant Material	FRAP μg TE/g d.w. Plant Material	H_2_O_2_ mg TE/g d.w. Plant Material	NO Scavenging Activity mg QE/g d.w. Plant Material
*A. absinthium* flowers (1 gr/1 mL)	87.54 ± 7.96	62.45 ± 4.33	85.18 ± 9.07	66.45 ± 5.16
*A. absinthium* leaves (1 gr/1.2 mL)	189.64 ± 16.09	46.45 ± 2.18	87.42 ± 6.24	145.12 ± 11.06
*A. annua* flowers (1 gr/1.2 mL)	73.08 ± 4.51	30.74 ± 1.25	70.59 ± 4.09	68.23 ± 3.92
*A.annua* leaves (1 gr/1.2 mL)	77.28 ± 4.71	51.43 ± 2.56	84.51 ± 4.75	64.93 ± 3.84
TROLOX (mg)	12.03 ± 0.44	11.26 ± 1.09	22.48 ± 1.73	
Quercitin (mg)				22.34 ± 2.48

Note: Values are expressed as mean ±SD (*n* = 3). DPPH—DPPH free radical scavenging activity; FRAP—ferric reducing antioxidant power; H_2_O_2_—hydrogen peroxide scavenging capacity; NO—nitric oxide radical scavenging assay; TE—TROLOX equivalent; QE—quercitin equivalent.

**Table 4:** In vitro cytotoxicity of A. absinthium and A. annua flower and leaf ethanolic extracts against human ovarian A2780cis, OVCAR-3, and OAW-42 tumor cells, and normal HaCaT cells; values expressed as the half inhibitory concentration (IC_50_, μg/mL); the values were expressed as mean ± SD from three independent experiments made in triplicates (*n* = 9).

Cell Line/ Plant Extract	A2780cis	OVCAR-3	OAW-42	HaCaT
*A. absinthium* flowers	13.96 ± 0.08	23.73 ± 1.14	21.82 ± 2.11	33.20 ± 0.86
*A. absinthium* leaf	18.48 ± 1.23	59.80 ± 3.85	28.06 ± 1.25	47.22 ± 0.30
*A. annua* flowers	9.29 ± 0.62	38.30 ± 2.99	16.24 ± 0.19	51.09 ± 1.88
*A.annua* leaf	15.70 ± 2.67	34.77 ± 0.81	18.45 ± 1.44	63.34 ± 1.76

**Table 5:** In vivo anti-inflammatory activity biomarkers of the study groups.

Groups	NfkB-p65 (ng/mL)	IL-1b (pg/mL)	IL-18 (pg/mL)	GSDMD (ng/mL)	Caspase-1 (pg/mL)
CONTROL	138.47 ± 8.52	22.36 ± 2.71	18.81 ± 7.12	4.91 ± 0.84	14.26 ± 1.34
INFL	382.05 ± 19.37 ^aaa^	58.25 ± 55.61 ^aaa^	60.09 ± 5.46 ^aaa^	10.14 ± 1.24 ^aaa^	139.08 ± 5.08 ^aaa^
AABF 100%	236.95 ± 12.88 ^b,cc,dd^	29.11 ± 5.50 ^bbb^	18.28 ± 1.93 ^bbb,cc^	5.08 ± 0.87 ^bbb^	19.08 ± 2.29 ^bbb,cc,dd^
AABF 50%	234.52 ± 25.73 ^b,cc,dd^	24.99 ± 2.55 ^bbb^	17.08 ± 1.06 ^bbb,cc^	6.00 ± 0.88 ^bbb^	32.23 ± 2.76 ^bbb^
AABF 25%	305.58 ± 20.42 ^ccc,ddd^	30.09 ± 6.31 ^bbb,ddd^	22.46 ± 2.70 ^bbb,c^	8.98 ± 0.74	39.89 ± 2.39 ^bbb^
AABL 100%	233.40 ± 43.43 ^b,cc,dd^	21.46 ± 3.76 ^bbb^	20.34 ± 4.99 ^bbb,c^	3.89 ± 0.64 ^bbb^	24.81 ± 3.71 ^bbb,cc,dd^
AABL 50%	269.33 ± 18.65 ^b,cc,dd^	26.02 ± 5.10 ^bbb^	22.91 ± 3.67 ^bbb,c^	4.87 ± 0.82 ^bbb^	36.30 ± 4.31 ^bbb^
AABL 25%	332.33 ± 18.31 ^ccc,ddd^	22.94 ± 2.56 ^bbb,ddd^	22.26 ± 4.23 ^bbb,c^	6.63 ± 0.12 ^b^	36.76 ± 3.00 ^bbb^
AANF 100%	256.51 ± 14.96 ^b,cc,dd^	27.35 ± 3.72 ^bbb^	18.51 ± 1.59 ^bbb,cc^	4.19 ± 0.61 ^bbb^	29.16 ± 4.89 ^bbb,cc,dd^
AANF 50%	241.39 ± 12.2 ^b,cc,dd^	27.61 ± 1.78 ^bbb^	18.54 ± 1.41 ^bbb,cc^	5.55 ± 0.49 ^bbb^	35.50 ± 4.05 ^bbb^
AANF 25%	239.89 ± 15.62 ^b,cc,dd^	24.22 ± 2.53 ^bbb^	22.23 ± 4.73 ^bbb,c^	9.15 ± 0.65	71.47 ± 7.24
AANL 100%	244.11 ± 20.12 ^b,cc,dd^	26.17 ± 4.18	16.47 ± 1.58 ^bbb,cc^	3.88 ± 0.44 ^bbb^	27.03 ± 3.86 ^bbb,cc,dd^
AANL 50%	253.23 ± 13.75 ^b,cc,dd^	24.46 ± 2.04 ^bbb^	18.74 ± 1.34 ^bbb,cc^	4.93 ± 0.42 ^bbb^	34.39 ± 3.54 ^bbb^
AANL 25%	260.91 ± 19.00 ^b,cc,dd^	28.13 ± 3.65 ^bbb^	24.51 ± 2.51 ^bbb,cc^	5.45 ± 0.26 ^bb^	73.26 ± 5.61
TX	149.51 ± 12.04 ^bbb^	25.49 ± 3.07 ^bbb^	31.65 ± 4.61 ^bbb^	5.98 ± 2.43 ^b^	38.31 ± 2.51 ^bbb^
DICLO	140.83 ± 11.65 ^bbb^	28.62 ± 2.53 ^bbb^	59.71 ± 5.16	5.42 ± 2.24 ^bb^	40.26 ± 2.82 ^bbb^

Note: Values are expressed as mean ± SD (*n* = 9). Vs CONTROL: ^aaa^ *p*  <  0.001; Vs INFL: ^b^ *p*  <  0.05; ^bb^ *p*  <  0.01; ^bbb^ *p*  <  0.001; Vs TX: ^c^ *p*  <  0.05; ^cc^ *p*  <  0.01; ^ccc^ *p*  <  0.001; Vs DICLO: ^dd^ *p*  <  0.01; ^ddd^ *p*  <  0.001; INFL—inflammation group; AABF 100%—*A. absinthium* flowers 100%; AABF 50%—*A. absinthium* flowers 50%; AABF 25%—*A. absinthium* flowers 25%; AABL 100%—*A. absinthium* leaves 100%; AABL 50%—*A. absinthium* leaves 50%; AABL 25%—*A. absinthium* leaves 25%; AANF 100%—*A. annua* flowers 100%; AANF 50%—*A. annua* flowers 50%; AANF 25%—*A. annua* flowers 25%; AANL 100%—*A. annua* leaves 100%; AALF 50%—*A. annua* leaves 50%; AALF 25%—*A. annua* leaves 25%; TX—Trolox; DICLO—diclofenac; NfkB-p65—Nuclear factor-κB; IL-1b—Interleukine 1-b; IL-18—Interleukine 18; GSDMD—Gasdermine D.

**Table 6:** In vivo antioxidant activity biomarkers of the study groups.

Groups	TOS (µmol H_2_O_2_E/L)	TAC (mmol TE/L)	OSI	AOPP (µmol/L)	MDA (nmol/L)	NO (µmol/L)	3-NT (ng/mL)	8-OhdG (ng/mL)	SH (µmol/L)
CONTROL	16.22 ± 4.16	1.08 ± 0.00	16.70 ± 3.46	26.93 ± 10.70	2.05 ± 0.14	19.22 ± 5.71	20.98 ± 2.75	21.07 ± 2.94	344.65 ± 30.18
INFL	47.81 ± 11.08 ^aaa^	1.06 ± 0.00 ^aaa^	43.09 ± 9.00 ^aaa^	68.28 ± 6.19 ^aaa^	5.55 ± 0.90 ^aaa^	67.00 ± 5.71 ^aaa^	63.22 ± 9.11 ^aaa^	87.64 ± 11.77 ^aaa^	220.78 ± 53.55 ^aaa^
AABF 100%	15.80 ± 2.45 ^bbb^	1.08 ± 0.00 ^bb,cc,dd^	18.19 ± 6.34 ^bbb^	21.42 ± 10.63 ^bbb^	3.00 ± 0.31 ^bb^	39.23 ± 17.22 ^bbb^	31.22 ± 7.19 ^bbb,cc^	22.89 ± 5.28 ^bbb,cc,dd^	653.25 ± 53.79 ^bbb,ccc,ddd^
AABF 50%	15.31 ± 4.90 ^bbb^	1.08 ± 0.00 ^bb,cc,dd^	12.13 ± 3.29 ^bbb^	31.03 ± 6.82 ^bbb^	3.09 ± 0.31 ^bb^	50.26 ± 5.60 ^bb,c,dd^	34.84 ± 1.43 ^bbb,cc^	27.57 ± 5.69 ^bbb,cc,dd^	524.75 ± 74.12 ^bbb,ccc,ddd^
AABF 25%	16.66 ± 4.41 ^bbb^	1.08 ± 0.00 ^bb,cc,dd^	16.85 ± 5.73 ^bbb^	35.01 ± 15.54 ^bbb^	3.32 ± 0.46 ^bb^	55.25 ± 5.88 ^bb,c,dd^	38.94 ± 3.33 ^bbb,c^	28.95 ± 9.42 ^bbb,cc,dd^	521.00 ± 66.57 ^bbb,ccc,ddd^
AABL 100%	19.67 ± 6.86 ^bbb^	1.08 ± 0.00 ^bb,cc,dd^	14.61 ± 2.27 ^bbb^	27.87 ± 6.62 ^bbb^	2.53 ± 0.12 ^bbb^	44.84 ± 7.88 ^bb,c,dd^	15.48 ± 8.72 ^bbb,dd^	21.50 ± 5.09 ^bbb,cc,dd^	642.71 ± 67.61 ^bbb,ccc,ddd^
AABL 50%	13.12 ± 3.56 ^bbb^	1.08 ± 0.00 ^bb,cc,dd^	14.16 ± 4.54 ^bbb^	22.83 ± 5.94 ^bbb^	2.54 ± 0.21 ^bbb^	44.43 ± 8.52 ^bb,c,dd^	31.47 ± 1.95 ^bbb,cc^	26.50 ± 8.62 ^bbb,cc,dd^	679.25 ± 85.87 ^bbb,ccc,ddd^
AABL 25%	18.22 ± 6.19 ^bbb^	1.08 ± 0.00 ^bb,cc,dd^	15.42 ± 4.08 ^bbb^	32.95 ± 18.11 ^bbb^	3.28 ± 0.80 ^bb^	47.57 ± 6.91 ^bb,c,dd^	32.75 ± 7.85 ^bb,cc^	37.40 ± 8.86 ^bbb,c,d^	571.25 ± 77.49 ^bbb,ccc,ddd^
AANF 100%	18.22 ± 2.57 ^bbb^	1.08 ± 0.00 ^bb,cc,dd^	9.27 ± 1.40 ^bbb^	27.24 ± 7.22 ^bbb^	2.67 ± 0.21 ^bbb^	34.61 ± 2.52 ^bbb,d^	12.48 ± 2.92 ^bbb,dd^	23.28 ± 1.26 ^bbb,cc,dd^	337.00 ± 56.10 ^bbb^
AANF 50%	23.60 ± 9.27 ^bbb^	1.08 ± 0.00 ^bb,cc,dd^	10.25 ± 2.28 ^bbb^	33.54 ± 14.62 ^bbb^	2.94 ± 0.25 ^bb^	34.16 ± 9.59 ^bbb,d^	14.99 ± 7.56 ^bbb,dd^	30.94 ± 7.05 ^bbb,c,d^	325.80 ± 68.45 ^bbb^
AANF 25%	21.11 ± 8.23 ^bbb^	1.08 ± 0.00 ^bb,cc,dd^	13.88 ± 4.13 ^bbb^	40.43 ± 13.83 ^bbb,cc,dd^	2.91 ± 0.25 ^bb^	35.96 ± 10.72 ^bbb,d^	29.03 ± 3.64 ^bbb,c^	34.05 ± 7.19 ^bbb,c,d^	328.60 ± 64.15 ^bbb^
AANL 100%	10.03 ± 1.51 ^bbb^	1.08 ± 0.00 ^bb,cc,dd^	16.85 ± 2.38 ^bbb^	20.71 ± 7.71 ^bbb^	3.15 ± 0.34 ^bb^	40.03 ± 18.42 ^bb,c,d^	18.14 ± 5.68 ^bbb,dd^	18.10 ± 2.01 ^bbb,ccc,ddd^	329.75 ± 41.60 ^bbb,c,d^
AANL 50%	11.02 ± 2.31 ^bbb^	1.08 ± 0.00 ^bb,cc,dd^	21.83 ± 8.57 ^bbb^	20.94 ± 7.11 ^bbb^	3.17 ± 0.32 ^bb^	60.60 ± 4.85 ^ccc,ddd^	24.95 ± 6.90 ^bbb,c^	20.40 ± 6.62 ^bbb,cc,dd^	281.00 ± 128.78 ^bb^
AANL 25%	15.00 ± 4.47 ^bbb^	1.08 ± 0.00 ^bb,cc,dd^	19.53 ± 7.61 ^bbb^	25.49 ± 6.63 ^bbb^	3.26 ± 0.41 ^bb^	64.95 ± 4.66 ^ccc,ddd^	27.30 ± 4.02 ^bbb,dd^	29.96 ± 6.32 ^bbb,cc,dd^	306.25 ± 73.26 ^bb^
TX	16.21 ± 4.82 ^bbb^	1.08 ± 0.00 ^bb^	15.24 ± 1.41 ^bbb^	26.22 ± 4.32 ^bbb^	2.28 ± 0.24 ^bbb^	35.66 ± 4.21 ^bbb^	31.24 ± 4.37 ^bbb^	40.06 ± 4.91 ^bbb^	301.08 ± 28.22 ^bb^
DICLO	18.27 ± 3.12 ^bbb^	1.08 ± 0.00 ^bb^	15.28 ± 1.32 ^bbb^	25.54 ± 3.65 ^bbb^	3.09 ± 0.95 ^bb^	22.09 ± 2.72 ^bbb^	34.41 ± 2.26 ^bbb^	48.12 ± 5.04 ^bbb^	297.13 ± 19.34

Note: Values are expressed as mean ± SD (*n* = 9). Vs CONTROL: ^aaa^ *p*  <  0.001; Vs INFL: ^bb^ *p*  <  0.01; ^bbb^ *p*  <  0.001; Vs TX: ^c^ *p*  <  0.05; ^cc^ *p*  <  0.01; ^ccc^ *p*  <  0.001; Vs DICLO: ^d^ *p* < 0.05; ^dd^ *p*  <  0.01; ^ddd^ *p*  <  0.001; INFL—inflammation group; AABF 100%—*A. absinthium* flowers 100%; AABF 50%—*A. absinthium* flowers 50%; AABF 25%—*A. absinthium* flowers 25%; AABL 100%—*A. absinthium* leaves 100%; AABL 50%—*A. absinthium* leaves 50%; AABL 25%—*A. absinthium* leaves 25%; AANF 100%—*A. annua* flowers 100%; AANF 50%—*A. annua* flowers 50%; AANF 25%—*A. annua* flowers 25%; AANL 100%—*A. annua* leaves 100%; AALF 50%—*A. annua* leaves 50%; AALF 25%—*A. annua* leaves 25%; TX—Trolox; DICLO—diclofenac; TOS—Total oxidative status; TAC—Total antioxidant capacity; OSI—Oxidative stress index; 8-OHdG—8-hydroxydeoxyguanosine; AOPP—Advanced oxidation protein products; MDA: Malonyldialdehide; NOx—Nitrites and nitrates; 3NT—3-nitrotyrosine; SH—total thiols.

**Table 7:** Liver and renal injury biomarkers of the study groups.

Groups	ALT	AST	Creatinine mg/dL	Ureea mg/dL
CONTROL	45.08 ± 9.17	48.62 ± 3.11	0.74 ± 0.02	32.22 ± 2.32
INFL	50.59 ± 4.82	49.21 ± 3.46	1.06 ± 0.03 ^aa^	54.43 ± 4.43
AABF 100%	48.04 ± 5.38	43.32 ± 3.65	0.93 ± 0.01	53.87 ± 6.46
AABF 50%	48.25 ± 4.90	43.19 ± 6.84	0.93 ± 0.06	52.08 ± 6.84
AABF 25%	47.90 ± 5.63	45.11 ± 2.31	0.94 ± 0.01	56.20 ± 6.69
AABL 100%	41.63 ± 4.33	45.43 ± 4.87	0.95 ± 0.09	50.29 ± 4.12
AABL 50%	46.08 ± 6.82	49.23 ± 4.05	0.92 ± 0.06	52.25 ± 3.84
AABL 25%	40.82 ± 3.02	49.71 ± 5.44	0.95 ± 0.08	51.60 ± 2.68
AANF 100%	49.55 ± 9.64	40.16 ± 2.35	0.97 ± 0.05	51.17 ± 6.80
AANF 50%	46.77 ± 4.15	49.74 ± 3.84	0.92 ± 0.08	41.49 ± 3.89
AANF 25%	46.55 ± 3.79	46.48 ± 5.64	0.87 ± 0.01	50.16 ± 8.91
AANL 100%	43.24 ± 2.02	40.10 ± 4.64	0.70 ± 0.02 ^bb^	32.98 ± 3.18 ^bb^
AANL 50%	39.59 ± 2.24	40.09 ± 4.95	0.75 ± 0.01 ^bb^	27.25 ± 3.45 ^bb^
AANL 25%	43.13 ± 4.98	47.20 ± 5.50	0.73 ± 0.02 ^bb^	34.06 ± 2.90 ^bb^
TX	32.13 ± 5.77	32.86 ± 2.74	0.74 ± 0.02 ^bb^	30.42 ± 2.22 ^bb^
DICLO	37.23 ± 2.61	35.80 ± 2.59	0.81 ± 0.02 ^bb^	47.48 ± 5.08 ^bb^

Note: Values are expressed as mean ± SD (*n* = 9). Vs CONTROL: ^aa^ *p*  <  0.01; Vs INFL: ^bb^ *p*  <  0.01; INFL—inflammation group; AABF 100%—*A. absinthium* flowers 100%; AABF 50%—*A. absinthium* flowers 50%; AABF 25%—*A. absinthium* flowers 25%; AABL 100%—*A. absinthium* leaves 100%; AABL 50%—*A. absinthium* leaves 50%; AABL 25%—*A. absinthium* leaves 25%; AANF 100%—*A. annua* flowers 100%; AANF 50%—*A. annua* flowers 50%; AANF 25%—*A. annua* flowers 25%; AANL 100%—*A. annua* leaves 100%; AALF 50%—*A. annua* leaves 50%; AALF 25%—*A. annua* leaves 25%; TX—Trolox; DICLO—diclofenac; ALT—alanin aminotransferase; AST—aspartat aminotransferase.

## Data Availability

Dataset available upon request from the authors.
